# The effects of sociodemographic factors and comorbidities on sepsis: A nationwide Swedish cohort study

**DOI:** 10.1016/j.pmedr.2023.102326

**Published:** 2023-07-16

**Authors:** Henning Stenberg, Xinjun Li, Wazah Pello-Esso, Sara Larsson Lönn, Sara Thønnings, Ardavan Khoshnood, Jenny Dahl Knudsen, Kristina Sundquist, Filip Jansåker

**Affiliations:** a Center for Primary Health Care Research, Department of Clinical Sciences Malmö, Lund University, Sweden; b Department of Clinical Microbiology, Copenhagen University Hospital Hvidovre, Denmark; c Department of Emergency Medicine, Skåne University Hospital Lund, Lund, Sweden; d Department of Clinical Microbiology, Center of Diagnostic Investigations, Rigshospitalet, Copenhagen University Hospital, Denmark; e Center for Community-based Healthcare Research and Education (CoHRE), Department of Functional Pathology, School of Medicine, Shimane University, Japan; f Department of Family Medicine and Community Health, Department of Population Health Science and Policy, Icahn School of Medicine at Mount Sinai, NY, USA

**Keywords:** Epidemiology, Risk factors, Sepsis, Severe mental disorders, Sociodemographic factors

## Abstract

Sepsis is a severe condition, representing a significant public health concern, especially in the elderly. There is, however, little insight into the potential effects of sociodemographic factors and comorbidities on sepsis incidence and how these factors interact. This was a nationwide open cohort study including individuals (N = 6 746 010) in Sweden ≥ 18 years of age spanning from 1997 to 2018, with 116 175 995 person years of follow-up. The outcome was time to first occurrence of sepsis. The following variables were included in the analysis: sociodemographic factors (age, sex, income, education, marital status, region of residency, and country of origin), severe mental disorders (schizophrenia and bipolar disorders), and Charlson Comorbidity Index. Interaction tests were conducted. A total of 161 558 individuals were diagnosed with sepsis during the study period, corresponding to an incidence rate of 13.9 per 10 000 person years (95% CI: 13.8 – 14.0). The main findings were that male sex, high age, low education, and comorbid conditions were positively associated with sepsis, after adjustments for the other covariates. Being aged 80 years and above yielded a HR of 18.19 (95% CI: 17.84 – 18.55) and the effect of high age was more than twice as high in men than in women. In conclusion, this large nationwide cohort found that several sociodemographic factors and comorbid conditions were independently associated with sepsis and men were more affected by higher age than women. These findings can help improve sepsis awareness and preventive work in risk groups.

## Highlights

• This study explores population-based risk factors for sepsis.
• The study utilized nationwide registers and primary healthcare data.
• High age and sociodemographic factors are associated with sepsis.
• The effect of high age is more than twice as high in men than in women.
• Somatic and mental disorders are also independently associated with sepsis.

## 1 Introduction

Sepsis is a disease of very high morbidity and mortality. (Reinhart et al., 2017, Rudd et al., 2020) It is caused by a dysregulated host response to a severe (bloodstream) infection, mainly bacterial, eventually leading to systemic inflammation and in many cases multiple organ dysfunction and death. (Singer et al., 2016) The mortality remains high even when treated (Rudd et al., 2020) and almost one in three deaths occurring in hospitals is estimated to be due to sepsis. (Rhee et al., 2017).

Older age and several somatic comorbidities, such as diabetes mellitus, cancer, and lung disease, are known risk factors for sepsis and severe bloodstream infections. (Martin et al., 2006, Laupland et al., 2004, Tsertsvadze et al., 2016, Koh et al., 2012, Global Burden of Disease Study C, 2015, Donnelly et al., 2018) Thus, ageing populations in combination with higher rates of chronic diseases (Global Burden of Disease Study C, 2015) could lead to an increased burden of sepsis on the health care system. In addition, little is known about which population-level risk factors may affect the risk of sepsis. Such studies are important as they may provide evidence for better preventive work in clinical practice, sepsis awareness in risk populations and risk reducing measures in the entire population.

Studies from the United States (US) (Martin et al., 2003, Mayr et al., 2010) and Canada (Hennessy et al., 2020) have identified male sex, minority background (e.g. African American ancestry) and low socioeconomic status (e.g. low income and/or education) as possible risk factors for sepsis. However, studies from other countries with different populations and healthcare systems as well as access to more comprehensive data sources have been warranted. (Hennessy et al., 2020, Rhee et al., 2015) Furthermore, although several reports on possible associations between various types of mental disorders and sepsis exist, findings are inconsistent. Some studies have shown an increased risk of sepsis in individuals with a history of mental disorder (Andersson et al., 2016) or a combination of mental and somatic diseases, such as cancer, (Liu et al., 2020) while others have found a lower mortality in patients with a wide range of mental disorders and hospitalization for sepsis. (Oud et al., 2022) A recently published Swedish study showed an increased risk of hospitalization and death due to sepsis over a span of two years in patients with severe mental disorders (psychotic or bipolar disorders), (Nilsson et al., 2021) but the results were not fully adjusted for sociodemographic factors and comorbidities.

In general, previous epidemiological studies on sepsis incidence seems mainly to have had a short follow-up period or used summarized regional data. Additionally, previous studies seem to have lacked the possibility of including largescale nationwide sociodemographic and clinical data (including from primary healthcare), precluding comprehensive explorations on sepsis (based on data with low levels of missing data and little loss to follow-up). Sweden, as a country with universal healthcare, has both nationwide healthcare records and general population registers of high completeness, with the possibility to link individual data from different registers via unique identification numbers, thus being an optimal setting to study these associations.

In this nationwide study, including the entire Swedish adult population and spanning 22 years, the main aim was to investigate the potential effects of sociodemographic factors and somatic as well as psychiatric comorbidities on sepsis incidence in addition to explore their interactions.

## 2 Materials and methods

### 2.1 Study design, population, and setting

A nationwide open-cohort study was conducted on all individuals ≥ 18 years of age during the period January 1, 1997, to December 31, 2018, in Sweden. Baseline characteristics were assessed in January 1997 or when an individual residing in Sweden reached ≥ 18 years of age. The STROBE-checklist for cohort studies was considered when conducting the study and writing the manuscript. The research was conducted at Lund University (Sweden).

### 2.2 Ascertainment of the outcome variables (sepsis)

The outcome was time to the first occurrence of a sepsis event during the study period. This was measured as the first sepsis (hospitalization) diagnosis obtained as both main and secondary diagnoses in the National Patient Register (NPR, described below) (Ludvigsson et al., 2011) and according to the Swedish adaptation of the World Health Organization’s 10th revision (second edition of the International Classification of Diseases (ICD-10). (Socialstyrelsen, 2018) Of all cases included, the majority were any of the two diagnoses A41 (“Other sepsis”; 81.2%) and A40 (“Sepsis due to streptococcus”; 13.2%), together compromising over 94.4% of all cases of sepsis in this study. The proportion of the rest of the sepsis diagnoses (5.6%) ranged from 0.0% to 2.5%. Supplementary Table S1 includes all sepsis ICD-10 diagnoses defining the outcome variable and number of cases included for each diagnosis. The table also include all the ICD-10 sepsis codes that were not considered in the analyses, e.g., the study did not include pregnancy-, pediatric/neonatal- and procedure (iatrogenic) -related sepsis diagnoses. These sepsis diagnoses were not considered as we aimed to study sepsis in adults not directly related to perinatal conditions and medical procedures. Moreover, the ICD-10 codes defining the outcome were used throughout the study period and the Sepsis-3 criteria (Singer et al., 2016) diagnoses were implemented in the ICD-10 diagnostic system in Sweden after the study period.

### 2.3 Ascertainment of sociodemographic variables / predictor variables

Sociodemographic (individual-level) factors were collected at baseline (i.e., at inclusion in the study) and defined as age, sex, family income, education, marital status, region of residency, and country of origin. *Age* groups were defined as 18–49, 50–59, 60–69, 70–79, or ≥ 80 years of age. *Sex* was defined as man or woman, measured as registered male or female sex. *Family income* was based on a weighted average income in each family and divided into quartiles where the two quartiles in the middle were collapsed: low (lowest quartile of the study population), middle (middle-low and middle-high quartiles) and high (highest quartile). *Education level* was classified into three different categories based on the duration of school years attended: compulsory education or less (≤9 years); some or completed high school education (10–12 years); at least one year of university or college education (≥12 years). Region of residence was categorized into three groups (i.e., residing in large cities, or outside of large cites in Southern- or Northern Sweden). *Marital status* was defined as married/cohabiting and unmarried/divorced/widowed. *Country of origin* was defined as: born in Sweden; Eastern Europe; Western countries; Middle East/North Africa (MENA); Africa (excluding North Africa); Asia (excluding the Middle East) and Oceania; or Latin America and the Caribbean.

### 2.4 Ascertainment of comorbidities

Comorbidities were assessed using diagnoses from ICD to 10. *Severe mental disorders* were measured as a diagnosis of any psychotic disorder (F20, F22, and F25) or bipolar/single manic episode (F30 and F31) during the study period. To take the possibility of confounding effects of other comorbidities into account we also included the Charlson Comorbidity Index (CCI) in the analysis. (Ludvigsson et al., 2021) The CCI, initially created to predict one year mortality, has also been used to predict other outcomes in observational studies. (Ludvigsson et al., 2021) The CCI includes several somatic chronic diseases and in this study the index was constructed based on CCI diagnoses codes from ICD to 9 (because the ICD classification was updated into ICD-10 in 1997). CCI diagnoses were measured within a five-year period prior to the 1997 baseline and used to categorize individuals into low [0p], medium [1–2p] and high [>2p] CCI-scores. The number of individuals with any of the CCI conditions as well as with and without sepsis are tabulated in Supplementary Table S2.

### 2.5 Data sources

The data sources were based on several nationwide registers with nearly complete coverage. All linkages between the registry data were performed using a pseudonymized version of the unique 10-digit personal identification number assigned to each person for their lifetime upon birth or immigration to Sweden. The register used to identify the outcome was the validated NPR, from which sepsis cases were identified from inpatient data (99% population coverage). The NPR (i.e. both inpatient- and outpatient data) and almost nationwide Swedish primary healthcare data (Sundquist et al., 2017) (including data from 20 of 21 administrative regions in Sweden) were used to identify comorbidities. The Total Population Register (complete population coverage for persons registered on 31 December, taking into account demographic events during the year) was used to collect data on the sociodemographic variables. This register was also used to track migration and vital statistics for individuals included in the study population. Individuals (n = 751 101) with missing observations on any sociodemographic variables were excluded from the main analysis, i.e., in total 10% of the population, but included in a sensitivity analysis.

### 2.6 Statistical analysis

Descriptive statistics on the study population, total person years of follow-up, number of first sepsis events, and incidence rates per 10 000 person years were calculated for each predictor variable with in total 116 175 995 person years of follow-up. Cox proportional hazards models were used to estimate the association between the predicting variables and the first sepsis event. The results are presented as hazard ratios (HR) and 95% confidence intervals (CI). The study period started on January 1, 1997, and individuals were followed from this date, the age of 18, or immigration (if 18 years or older at the time) to Sweden until an outcome event, death, emigration, or end of the study period (December 31, 2018), whichever came first. Three models were used: Model 1, a univariable model; Model 2, a model also adjusted for age; and Model 3, the full model, including all covariates. Furthermore, we evaluated whether the impact of the covariates on the first sepsis event varied by sex by including interaction terms between sex and each of the covariates in Model 3. We also performed sensitivity analyses (Table S3–S5). We tested the proportional hazard assumptions by plotting the incidence rates over time and by calculating Schoenfeld (partial) residuals and these assumptions were fulfilled. A two-tailed p-value of < 0.05 was used to determine statistical significance. SAS software version 9.4 (SAS Institute Inc.; Cary, NC, USA) was used for all statistical analyses.

Ethical statement

The present study was a non-intervention nationwide register study based on pseudonymized secondary data collected by and obtained from Swedish authorities and approved for use by the Ethical Review Board in Lund, Sweden.

## 3 Results

**Fig. 1:**
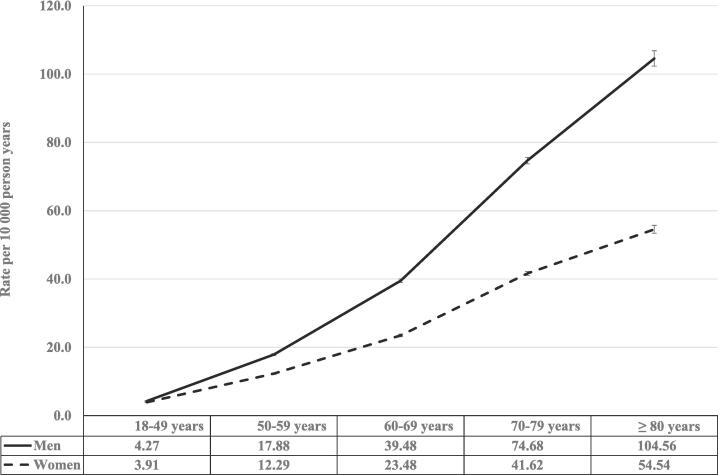
Age-specific incidence rates per 10 000 person years (first episode in the study period) of sepsis in adults in Sweden by sex (1997–2018). Legend: The table shows the incidence rates (per 10 000 person-years, including 95% confidence interval) of the first episode of sepsis in adults in Sweden during the study period 1997-2018. The incidence rates are stratified by age-groups and by sex.

Table 1 shows the descriptive statistics of the study population which consisted of 6 746 010 individuals of which 161 558 were diagnosed with sepsis during the study period. The follow-up was 116 175 995 person years and the incidence rate of the first sepsis event was 13.9 (95% CI: 13.8 – 14.0) per 10 000 person years. The incidence rate was 15.7 (95% CI: 15.6 – 15.8) in men and 12.1 (95% CI: 12.0 – 12.2) in women. Age-specific incidence rates varied between 4.1 (95% CI: 4.0 – 4.2) for the age group 18–49 years and rose steeply to the highest incidence rates in the ≥ 80 years age category of 71.3 (95% CI: 70.2 – 72.4). Individuals with the lowest educational level (≤9 years) had an incidence rate of 22.8 (95% CI: 22.6 – 22.9) compared to 10.2 (95% CI: 10.1 – 10.3) for those with the highest educational level (>12 years). In contrast, individuals with low family income had lower incidence rate (11.2, 95% CI: 11.0 – 11.3) than those with moderate and high family income. For the covariates region of residence and marital status, individuals living in large cities (14.8, 95% CI: 14.7 – 14.9) and married/cohabiting (16.5, 95% CI: 16.4 – 16.6) had the highest incidence rates. For country of origin, the incidence rates varied between 6.1 (Africa, excluding North Africa) and 17.4 (Western countries). Individuals with severe mental disorders had higher incidence rates (16.2, 95% CI: 15.6 – 16.7) than those without (13.9 95% CI: 13.8 – 13.9). Particularly high incidence rates were observed for those with moderate (61.1, 95% CI: 60.4 – 61.9) and high (80.8, 95% CI: 62.4 – 99.2) scores in the CCI. Fig. 1 shows the age-specific incidence rates by sex during the follow-up. Men had higher incidence rates of sepsis than women in all age-groups and the curve for the men was also steeper with increasing age.

**Table 1:** Study population of adults, number of sepsis cases, and incidence rates of sepsis in Sweden (1997–2018).

	Total population		Events of sepsis		Incidence rate per 10 000 person years		
	No.	%	No.	%	IR	95% CI	
**Total population**	6 746 010		161 558		13.9	13.8	14.0
**Sex**							
Men	3 294 949	48.8	90 439	56.0	15.7	15.6	15.8
Women	3 451 061	51.2	71 119	44.0	12.1	12.0	12.2

**Age** at baseline (years)							
18–49	3 706 719	54.9	29 605	18.3	4.1	4.0	4.1
50–59	1 113 444	16.5	31 318	19.4	15.1	14.9	15.3
60–69	783 999	11.6	39 152	24.2	30.9	30.6	31.2
70–79	716 588	10.6	44 823	27.7	55.0	54.5	55.5
≥ 80	425 260	6.3	16 660	10.3	71.3	70.2	72.4

**Educational level** (years)							
≤ 9	2 170 482	32.2	76 817	47.5	22.8	22.6	22.9
10–12	2 883 602	42.7	54 959	34.0	10.3	10.2	10.4
>12	1 691 926	25.1	29 782	18.4	10.2	10.1	10.3

**Family income** (quartile)							
Low	1 681 157	24.9	31 655	19.6	11.2	11.0	11.3
Middle	3 375 078	50.0	87 867	54.4	15.4	15.3	15.5
High	1 689 775	25.0	42 036	26.0	13.6	13.5	13.8

**Region of residence**							
Large cities	3 311 976	49.1	84 303	52.2	14.8	14.7	14.9
Southern Sweden	2 296 973	34.0	50 714	31.4	12.8	12.7	12.9
Northern Sweden	1 137 061	16.9	26 541	16.4	13.6	13.4	13.8

**Marital status**							
Married/Cohabiting	3 144 475	46.6	90 462	56.0	16.5	16.4	16.6
Unmarried/Divorced/Widowed	3 601 535	53.4	71 096	44.0	11.6	11.5	11.7

**Country of origin** (immigration status)							
Sweden (born in)	5 979 590	88.6	145 658	90.2	14.1	14.0	14.2
Eastern Europe	187 058	2.8	3782	2.3	11.6	11.2	12.0
Western countries	336 902	5.0	9276	5.7	17.4	17.1	17.8
Middle East/North Africa	106 465	1.6	1251	0.8	6.5	6.1	6.8
Africa (excluding North Africa)	26 851	0.4	273	0.2	6.1	5.4	6.8
Asia (excluding Middle East) and Oceania	69 731	1.0	875	0.5	7.0	6.5	7.4
Latin America and the Caribbean	39 413	0.6	443	0.3	6.4	5.8	7.0

**Charlson Comorbidity Index**							
Low (0p)	6 306 568	93.5	134 274	83.1	12.0	12.0	12.1
Moderate (1–2p)	438 512	6.5	27,210	16.8	61.1	60.4	61.9
High (>2p)	930	0.0	74	0.0	80.8	62.4	99.2

**Severe mental disorders**							
No diagnosis	6 646 796	98.5	158 660	98.2	13.9	13.8	13.9
Diagnosis	99 214	1.5	2898	1.8	16.2	15.6	16.7

IR: Incidence rate (of the first episode of sepsis in the follow-up period); CI: Confidence interval.

Table 2 shows the three models of the association between the predicting variables and the first sepsis event. Model 1, the univariable analyses, shows that all the included variables were significantly associated with a first sepsis event. Age and high CCI scores were strongly associated with sepsis. Being aged 80 years and above yielded a HR of 18.19 (95% CI: 17.84 – 18.55) compared to the reference age group 18–49 years. Individuals with the highest CCI scores had a HR of 6.79 (95% CI: 5.41 – 8.52). Men had a higher risk of sepsis (HR = 1.34, 95% CI: 1.32 – 1.35) than women and those with the lowest education level had a HR of 2.20 (95% CI: 2.17 – 2.23). The risks of a first sepsis event were lower in almost all immigrant groups compared to the Swedish-born reference group. In Model 2, following adjustment for age, the HR for men increased to 1.64 (95% CI: 1.63 – 1.66) whereas the HR for low education decreased to 1.19 (95% CI: 1.17 – 1.20). The HRs of a first sepsis event increased in almost all immigrant groups and became close to the HR of 1 compared to the reference group. Among individuals with the highest CCI scores the HR decreased to 4.23 (95% CI: 3.37 – 5.31). In Model 3, the full model, most of the results from Model 2 remained almost unaltered.

**Table 2:** Association of individual sociodemographic factors, comorbidities, and sepsis in adults in Sweden (1997–2018).

	Model 1			Model 2			Model 3		
Covariates	HR	95% CI		HR	95% CI		HR	95% CI	
**Age** (ref. age 18–49 years)									
50–59	3.69	3.63	3.75	3.69	3.63	3.75	3.62	3.56	3.68
60–69	7.56	7.45	7.68	7.56	7.45	7.68	7.11	6.99	7.22
70–79	13.83	13.62	14.03	13.83	13.62	14.03	12.46	12.26	12.67
≥ 80	18.19	17.84	18.55	18.19	17.84	18.55	16.35	16.01	16.69

**Sex** (ref. women)	1.34	1.32	1.35	1.64	1.63	1.66	1.64	1.62	1.65

**Educational level** (ref. > 12 years)									
≤ 9	2.20	2.17	2.23	1.19	1.17	1.20	1.18	1.16	1.19
10–12	1.01	1.00	1.02	1.07	1.05	1.08	1.05	1.04	1.07

**Family income** (ref. High)									
Low	0.82	0.80	0.83	1.04	1.02	1.06	1.06	1.04	1.07
Middle	1.12	1.11	1.13	1.08	1.06	1.09	1.09	1.07	1.10

**Region of residence** (ref. Large cities)									
Southern Sweden	0.86	0.85	0.87	0.82	0.81	0.83	0.80	0.79	0.80
Northern Sweden	0.92	0.91	0.93	0.87	0.85	0.88	0.83	0.82	0.84

**Marital status** (ref. Married/cohabiting)	0.71	0.70	0.71	0.98	0.97	0.99	1.03	1.02	1.04

**Country of Origin** (ref. Sweden)									
Eastern Europe	0.82	0.80	0.85	0.97	0.94	1.00	0.97	0.93	1.00
Western countries	1.23	1.21	1.26	1.06	1.04	1.08	1.06	1.04	1.08
Middle East/North Africa	0.46	0.44	0.49	0.97	0.92	1.02	0.91	0.86	0.96
Africa (excluding North Africa)	0.43	0.39	0.49	1.11	0.99	1.25	1.04	0.92	1.17
Asia/Oceania (excluding Middle East)	0.50	0.47	0.53	0.98	0.92	1.05	0.97	0.90	1.03
Latin America/Caribbean	0.45	0.41	0.50	0.86	0.79	0.95	0.84	0.76	0.92

**Charlson Comorbidity Index** (ref. Low)									
Moderate (1–2p)	5.06	5.00	5.13	2.33	2.30	2.36	2.27	2.24	2.30
High (>2p)	6.79	5.41	8.52	4.23	3.37	5.31	4.06	3.23	5.10

**Severe mental disorders** (ref. No diagnosis)	1.16	1.12	1.21	1.62	1.56	1.68	1.65	1.59	1.71

Model 1: Univariable models; Model 2: Age adjusted univariable models; Model 3: Fully adjusted for all covariates. HR: Hazard ratio; CI: Confidence interval.

In Table 3, we examined whether the associations between sepsis and the covariates differed by sex. The ratio between men and women showed that the association between age and sepsis was more pronounced in men than in women with an approximately two-fold increased risk in the oldest group for men (ratio: 2.09, 95% CI: 2.01 – 2.18) compared to women. There was also evidence that the association between sepsis and country of origin differed by sex. Most notably, among those originating from Africa (excluding North Africa) the ratio was 0.70 (95% CI: 0.55 – 0.89), indicating that the association between originating from Africa and sepsis was less pronounced in men than in women. Similar results of a weaker association between country of origin and sepsis in men was observed for almost all other immigrant groups; ratios varied between 0.83 and 0.93 and most of these ratios were statistically significant. We also performed sensitivity analyses, i.e., excluding those aged 15–49 years (Table S3), applying a shorter follow-up 1997–2002 (Table S4), and estimating our main results without any exclusions of those with missing data (Table S5). Most of the main results remained in all these sensitivity analyses.

**Table 3:** Association and interaction test of individual sociodemographic variables and sepsis by sex, in adults in Sweden (1997–2018).

	Men			Women			Ratio between men and women		
Covariates	HR	95% CI		HR	95% CI		Ratio	95% CI	
**Age** (ref. age 18–49 years)									
50–59	4.13	4.04	4.22	3.09	3.02	3.17	1.33	1.29	1.38
60–69	8.73	8.54	8.93	5.43	5.30	5.57	1.61	1.56	1.66
70–79	16.15	15.80	16.52	8.97	8.76	9.18	1.80	1.74	1.86
≥ 80	23.16	22.51	23.84	11.06	10.74	11.39	2.09	2.01	2.18

**Educational level** (ref. > 12 years)									
≤ 9	1.13	1.11	1.16	1.27	1.24	1.30	0.89	0.87	0.92
10–12	1.03	1.01	1.04	1.10	1.07	1.12	0.93	0.91	0.96

**Family income** (ref. High)									
Low	1.04	1.01	1.06	1.08	1.06	1.11	0.96	0.93	0.99
Middle	1.06	1.04	1.07	1.13	1.10	1.15	0.94	0.91	0.96

**Region of residence** (ref. Large cities)									
Southern Sweden	0.78	0.77	0.79	0.82	0.80	0.83	0.96	0.93	0.98
Northern Sweden	0.82	0.80	0.83	0.84	0.83	0.86	0.97	0.94	0.99

**Marital status** (ref. Married/cohabiting)	1.05	1.03	1.06	1.08	1.06	1.10	0.97	0.95	0.99

**Country of Origin** (ref. Sweden)									
Eastern Europe	0.93	0.89	0.97	1.01	0.96	1.06	0.92	0.87	0.99
Western countries	1.06	1.03	1.10	1.07	1.04	1.10	1.00	0.96	1.04
Middle East/North Africa	0.86	0.80	0.93	1.04	0.95	1.13	0.83	0.74	0.93
Africa (excluding North Africa)	0.90	0.76	1.06	1.27	1.08	1.51	0.70	0.55	0.89
Asia/Oceania (excluding Middle East)	0.88	0.80	0.97	1.04	0.95	1.14	0.85	0.74	0.97
Latin America/Caribbean	0.81	0.71	0.93	0.87	0.76	0.99	0.93	0.77	1.12

**Charlson Comorbidity Index** (ref. Low)									
Moderate (1–2p)	2.10	2.06	2.13	2.46	2.41	2.51	0.85	0.83	0.87
High (>2p)	4.31	3.25	5.72	3.58	2.44	5.26	0.85	0.83	0.87

**Severe mental disorders** (ref. No diagnosis)	1.59	1.51	1.68	1.68	1.60	1.77	0.94	0.88	1.02

Fully adjusted for all covariates. HR: Hazard Ratio; CI: Confidence interval.

## 4 Discussion

This nationwide cohort study included over 6.7 million adults with over 116-million person years of 22-year follow-up in order to examine several risk factors for the first sepsis occurrence. Male sex, high age, low education, and comorbid conditions were positively associated with sepsis, after adjustments for the other covariates. Being aged 80 years and above yielded a HR of 18.19 (95% CI, 17.84 – 18.55) and the effect of high age was more than twice as high in men than in women.

There are several possible explanations and potential mechanisms behind our findings. Firstly, old age is a well-known risk factor for sepsis. (Martin et al., 2006, Tsertsvadze et al., 2016, Rowe and McKoy, 2017) The increased risk of sepsis by higher age is likely due to impairments in the immune response which both leads to a higher probability to develop an infection (Rowe and McKoy, 2017) and an increased risk that the infection advances to sepsis. (Castle et al., 2007) Besides, it is also possible that older people suffer diagnostic delay due to more atypical clinical presentations. (Clifford et al., 2016) As for the association between sex and sepsis, several mechanisms might explain the increased risk of sepsis in men compared to women, such as the effect of sex hormones on the immune response, inflammation, and cardiovascular system. (Angele et al., 2014, Bosch et al., 2018) For example, male sex hormones have been shown to suppress certain cell-mediated immune responses while the opposite is true for female sex hormones. (Angele et al., 2014) Moreover, proinflammatory cytokines possibly contributing to the dysregulated host response, e.g. IL-6 and procalcitonin, have been found to be elevated in male patients compared to female patients with severe injury, and the incidence of post-traumatic sepsis is higher in men even when the extent of the predisposing injury is similar. (Oberholzer et al., 2000, Frink et al., 2007) It is possible that these mechanisms could interact, causing the positive association between age and sepsis to be more pronounced in men compared to women, as seen in the present study, which, to our knowledge, has not been comprehensively studied before. All this considered, the immunosenescence (Castle et al., 2007) and certain female biological characteristics (Angele et al., 2014, Bosch et al., 2018) could be part of the mechanisms behind the lower vulnerability in elderly women to acquire sepsis.

Previous studies from the US have found Afro-Americans to be at higher risk of sepsis compared to white individuals, but without any accounts of sex differences. (Mayr et al., 2010) In the present study, the risk for sepsis was not strongly associated with country of origin, after adjustments for the other covariates. However, men from Africa (excluding North Africa) seemed to be less influenced by their country of origin than African women. Similar results of a weaker association between country of origin and sepsis in men were observed for almost all other immigrant groups. Several possible explanations behind these differences might exist, such as sex differences between immigrants regarding integration and health status; these may include differences in knowledge and utilization of healthcare, including dissimilarities in the use of prevention and treatment for infectious diseases.

It is well known that severe mental disorder is linked to increased mortality overall, and several factors have been suggested to contribute to this, such as an unhealthy lifestyle, lower health literacy and under-diagnosis and under-treatment of somatic disorders. (Crump et al., 2013, Crump et al., 2013) We have shown that severe mental disorder also constitutes an independent risk factor for sepsis. We were unable to examine the reasons behind these findings, but healthcare seeking patterns or differential distribution in comorbidities might be possible explanations.

Previous studies have suggested an association between low socioeconomic status and sepsis, (Martin et al., 2003, Mayr et al., 2010, Hennessy et al., 2020) although studies from other settings and with more comprehensive data have been warranted. (Hennessy et al., 2020, Rhee et al., 2015) In this present study, low educational level and income was associated with sepsis incidence. However, low income was inversely associated with sepsis in the univariable model in contrast to education. However, after adjusting for age the inverse association for income became positive. This is most likely because income is generally lower for younger adults, which is in contrast to educational level and the association between educational level and sepsis was also attenuated after adjusting for age. The impact of low socioeconomic status on sepsis risk could be attributed to various factors, such as limited access to healthcare, unhealthy lifestyles, and low adherence to treatment for comorbidities. Notably, we observed a difference in the association between socioeconomic status and sepsis when comparing men and women. This could possibly be attributed to variations in the underlying mechanisms (Angele et al., 2014, Bosch et al., 2018) between men and women across socioeconomic strata.

The study has some limitations that needs to be considered. Firstly, as most other large-scale register studies, another limitation was that we were unable to include detailed clinical data. (Rhee et al., 2015) Secondly, there is a possibility that some cases of other severe medical conditions were misdiagnosed as sepsis whereas some cases of sepsis might be missed. A recent *meta*-analysis has indicated that under-coding of sepsis exists, (Liu et al., 2022) which introduces a potential bias stemming from missed cases of sepsis in research based on secondary data. However, it is unclear whether the degree of misclassification varies across different settings and population groups. Such variation may, however, result in a conservative bias. For example, it is possible that sepsis diagnoses were more likely to be missed in risk populations (e.g. elderly) (Rowe and McKoy, 2017). This would mean that the magnitudes of the HRs associated with sepsis in the present study are underestimated. We performed a sensitivity analysis excluding the youngest age category (individuals aged 15–49 years), and the results remained almost unchanged for all other variables. It is also important to consider that the sociodemographic factors were assessed at baseline, and they could have changed during the study period. We conducted a sensitivity analysis to partly address this, applying a shorter (six years) follow-up, in which the results were almost unchanged. Lastly, we used a relatively crude measure on region of residence and were unable to identify any large effects of this variable. A German study (Rose et al., 2021) showed that the number of hospital beds or general practitioners in the region had no effect on sepsis incidence, but the incidence increased with a longer distance to the nearest pharmacy. Although not measured in our study, the distance to the nearest pharmacy would most likely be shorter for those residing in large cities and, if so, the results regarding region of residency may differ between studies. (Rose et al., 2021).

There are also several strengths with the present study. Firstly, it included the entire adult population of Sweden. It spans a long time period and has access to detailed individual-level sociodemographic and medical information from highly reliable sources with high completeness for the entire population and little loss to follow-up. The ability to link clinical diagnoses to sociodemographic variables provides a unique opportunity to identify subpopulations at increased risk of sepsis. Secondly, many previous studies lack comprehensive data on comorbidities which may be important confounders. We were able to address this by our access to nationwide healthcare data from both hospital and primary healthcare settings where we constructed a more complete CCI than generally possible. Furthermore, several of our findings were in line with previous studies, which shows that our findings are robust. For example, the incidence rates of sepsis in Sweden during the study period was similar to previously published results from countries with similar healthcare. (Rose et al., 2021) This makes our findings more generalizable to other countries of similar healthcare systems. High age and high comorbidity scores were the main risk factors for sepsis, which is in line with previous research.^5-10^ Finally, the present study includes hitherto lacking nationwide primary healthcare data, which increased the coverage of comorbidities. (Sundquist et al., 2017) Altogether, this suggest that our findings are valid and that the registers applied in this study can be used to identify sepsis on a nationwide level.

With an increasing life expectancy and an ageing population with increased prevalence of chronic diseases, (Global Burden of Disease Study C, 2015) the incidence of sepsis is expected to rise. Increased knowledge of potential risk factors, which can be provided through public health campaigns and educational initiatives, is therefore of importance to improve the preventive work and awareness of sepsis in the entire populations and those at greater risk. Additional preventive strategies can include enhancing the access to primary healthcare among high-risk populations, improving vaccinations for pathogens associated with sepsis, and promoting regular health check-ups in the elderly population to prevent, detect, and manage sepsis risk factors. However, preventive measures should be tailored and resources allocated in an evidence-based and cost-effective manner, considering that sepsis is a relatively rare disease.

## 5 Conclusions

Sociodemographic factors and somatic and mental disorders are independently associated with sepsis. Clinicians could use this information for a higher awareness of sepsis in risk populations. Further research exploring the potential mechanisms behind our findings are warranted.

## Funding

This work was supported by non-commercial Swedish research funding granted to Filip Jansåker: i.e., by governmental funding of clinical research within the National Health Services, Region Skåne, Sweden (ALF-YF, 2022–0071); the Swedish Society of Medicine (SLS-960562, SLS-960574); Thelma Zoegas foundation (TZ2021-0003) and Maggie Stephens Stiftelse (20212001). The funding sources of the study were all non-commercial from Sweden and had no role in the study design; the collection, analysis, and interpretation of data; the writing of the report; or in the decision to submit the paper for publication.

## CRediT authorship contribution statement

All authors have approved the final version of the manuscript. Concept: FJ. Development of idea and design: FJ, AK, and KS. Critical revision and approval of design: All authors. Funding: FJ. Access and acquisition of data: KS. Analysis and statistics: XL, WPE and SLL. Tables: XL, FJ, SLL, WPE, and HS. Interpretation of data: All authors. Literature search: FJ, ST, HS, JDK, and WPE. Drafting of manuscript: HS, WPE, and FJ. Critical revision of the manuscript for intellectual content: All authors. The authors attest that all listed authors meet the authorship criteria and that no others meeting the criteria have been omitted.

## Declaration of Competing Interest

The authors declare that they have no known competing financial interests or personal relationships that could have appeared to influence the work reported in this paper.

## Data Availability

The authors do not have permission to share data.

## References

[b0005] Andersson N.W., Goodwin R.D., Okkels N., Gustafsson L.N., Taha F., Cole S.W., Munk-Jørgensen P. (2016). Depression and the risk of severe infections: prospective analyses on a nationwide representative sample. Int. J. Epidemiol..

[b0010] Angele M.K., Pratschke S., Hubbard W.J., Chaudry I.H. (2014). Gender differences in sepsis: cardiovascular and immunological aspects. Virulence.

[b0015] Bosch F., Angele M.K., Chaudry I.H. (2018). Gender differences in trauma, shock and sepsis. Mil. Med. Res..

[b0020] Castle S.C., Uyemura K., Fulop T., Makinodan T. (2007). Host Resistance and Immune Responses in Advanced Age. Clin. Geriatr. Med..

[b0025] Clifford K.M., Dy-Boarman E.A., Haase K.K., Maxvill K., Pass S.E., Alvarez C.A. (2016). Challenges with diagnosing and managing sepsis in older adults. Expert Rev. Anti Infect. Ther..

[b0030] Crump C., Winkleby M.A., Sundquist K., Sundquist J. (2013). Comorbidities and mortality in persons with schizophrenia: a Swedish national cohort study. Am. J. Psychiatry.

[b0035] Crump C., Sundquist K., Winkleby M.A., Sundquist J. (2013). Comorbidities and mortality in bipolar disorder: a Swedish national cohort study. JAMA Psychiat..

[b0040] Donnelly JP, Lakkur S, Judd SE, et al. (2018) Association of Neighborhood Socioeconomic Status With Risk of Infection and Sepsis. Clin Infect Dis 66(12): 1940-1947. 10.1093/cid/cix1109. 10.1093/cid/cix1109 PMC6248765 29444225

[b0045] Frink M., Pape H.C., van Griensven M., Krettek C., Chaudry I.H., Hildebrand F. (2007). Influence of sex and age on mods and cytokines after multiple injuries. Shock.

[b0050] Global Burden of Disease Study C (2015). Global, regional, and national incidence, prevalence, and years lived with disability for 301 acute and chronic diseases and injuries in 188 countries, 1990–2013: a systematic analysis for the Global Burden of Disease Study 2013. Lancet.

[b0055] Hennessy D.A., Soo A., Niven D.J., Jolley R.J., Posadas-Calleja J., Stelfox H.T., Doig C.J. (2020). Socio-demographic characteristics associated with hospitalization for sepsis among adults in Canada: a Census-linked cohort studyCaractéristiques sociodémographiques associées à l’hospitalisation suite à un sepsis chez les adultes au Canada : une étude de cohorte liée au Recensement. Can. J. Anaesth..

[b0060] Koh G.C., Peacock S.J., van der Poll T., Wiersinga W.J. (2012). The impact of diabetes on the pathogenesis of sepsis. Eur. J. Clin. Microbiol. Infect. Dis..

[b0065] Laupland K.B., Gregson D.B., Zygun D.A., Doig C.J., Mortis G., Church D.L. (2004). Severe bloodstream infections: a population-based assessment. Crit. Care Med..

[b0070] Liu Q, Song H, Andersson TM, et al. (2020) Psychiatric Disorders Are Associated with Increased Risk of Sepsis Following a Cancer Diagnosis. Cancer Res 80(16): 3436-3442. 10.1158/0008-5472.CAN-20-0502. 10.1158/0008-5472.CAN-20-0502 32532824

[b0075] Liu B., Hadzi-Tosev M., Liu Y., Lucier K.J., Garg A., Li S., Heddle N.M., Rochwerg B., Ning S. (2022). Accuracy of international classification of diseases, 10th revision codes for identifying sepsis: a systematic review and meta-analysis. Crit Care Explor.

[b0080] Ludvigsson J.F., Andersson E., Ekbom A., Feychting M., Kim J.-L., Reuterwall C., Heurgren M., Olausson P.O. (2011). External review and validation of the Swedish national inpatient register. BMC Public Health.

[b0085] Ludvigsson J.F., Appelros P., Askling J., Byberg L., Carrero J.-J., Ekström A.M., Ekström M., Smedby K.E., Hagström H., James S., Järvholm B., Michaelsson K., Pedersen N.L., Sundelin H., Sundquist K., Sundström J. (2021). Adaptation of the Charlson comorbidity index for register-based research in Sweden. Clin. Epidemiol..

[b0090] Martin G.S., Mannino D.M., Eaton S., Moss M. (2003). The epidemiology of sepsis in the United States from 1979 through 2000. N. Engl. J. Med..

[b0095] Martin G.S., Mannino D.M., Moss M. (2006). The effect of age on the development and outcome of adult sepsis. Crit. Care Med..

[b0100] Mayr F.B., Yende S., Linde-Zwirble W.T. (2010). Infection rate and acute organ dysfunction risk as explanations for racial differences in severe sepsis. J. Am. Med. Assoc..

[b0105] Nilsson N.H., Bendix M., Ohlund L., Widerstrom M., Werneke U., Maripuu M. (2021). Increased risks of death and hospitalization in influenza/pneumonia and sepsis for individuals affected by psychotic disorders, bipolar disorders, and single manic episodes: a retrospective cross-sectional study. J. Clin. Med..

[b0110] Oberholzer A., Keel M., Zellweger R., Steckholzer U., Trentz O., Ertel W. (2000). Incidence of septic complications and multiple organ failure in severely injured patients is sex specific. J. Trauma.

[b0115] Oud L., Garza J., Ehrman R. (2022). Impact of history of mental disorders on short-term mortality among hospitalized patients with sepsis: a population-based cohort study. PLoS One.

[b0120] Reinhart K., Daniels R., Kissoon N., Machado F.R., Schachter R.D., Finfer S. (2017). Recognizing sepsis as a global health priority - a WHO resolution. N. Engl. J. Med..

[b0125] Rhee C., Murphy M.V., Li L., Platt R., Klompas M. (2015). Comparison of trends in sepsis incidence and coding using administrative claims versus objective clinical data. Clin. Infect. Dis..

[b0130] Rhee C., Dantes R., Epstein L., Murphy D.J., Seymour C.W., Iwashyna T.J., Kadri S.S., Angus D.C., Danner R.L., Fiore A.E., Jernigan J.A., Martin G.S., Septimus E., Warren D.K., Karcz A., Chan C., Menchaca J.T., Wang R., Gruber S., Klompas M. (2017). Incidence and trends of sepsis in US hospitals using clinical vs claims data, 2009–2014. J. Am. Med. Assoc..

[b0135] Rose N., Matthäus-Krämer C., Schwarzkopf D., Scherag A., Born S., Reinhart K., Fleischmann-Struzek C. (2021). Association between sepsis incidence and regional socioeconomic deprivation and health care capacity in Germany - an ecological study. BMC Public Health.

[b0140] Rowe T.A., McKoy J.M. (2017). Sepsis in older adults. Infect. Dis. Clin. North Am..

[b0145] Rudd K.E., Johnson S.C., Agesa K.M., Shackelford K.A., Tsoi D., Kievlan D.R., Colombara D.V., Ikuta K.S., Kissoon N., Finfer S., Fleischmann-Struzek C., Machado F.R., Reinhart K.K., Rowan K., Seymour C.W., Watson R.S., West T.E., Marinho F., Hay S.I., Lozano R., Lopez A.D., Angus D.C., Murray C.J.L., Naghavi M. (2020). Global, regional, and national sepsis incidence and mortality, 1990–2017: analysis for the global burden of disease study. Lancet.

[b0150] Singer M., Deutschman C.S., Seymour C.W., Shankar-Hari M., Annane D., Bauer M., Bellomo R., Bernard G.R., Chiche J.-D., Coopersmith C.M., Hotchkiss R.S., Levy M.M., Marshall J.C., Martin G.S., Opal S.M., Rubenfeld G.D., van der Poll T., Vincent J.-L., Angus D.C. (2016). The third international consensus definitions for sepsis and septic shock (sepsis-3). J. Am. Med. Assoc..

[b0155] Socialstyrelsen (2018) *Internationell statistisk klassifikation av sjukdomar och relaterade hälsoproblem – Systematisk förteckning, svensk version 2018 (ICD-10-SE).* In English: International Statistical Classification of Diseases and Related Health Problems, Tenth Revision (ICD-10), Swedish version 2018 (ICD-10-SE). By the Swedish National Board of Health and Welfare (Socalstyrelsen). ISBN: 978-91-7555-599-7. www.socialstyrelsen.se Last accessed 2023-06-09.

[b0160] Sundquist J., Ohlsson H., Sundquist K., Kendler K.S. (2017). Common adult psychiatric disorders in Swedish primary care where most mental health patients are treated. BMC Psychiatry.

[b0165] Tsertsvadze A., Royle P., Seedat F., Cooper J., Crosby R., McCarthy N. (2016). Community-onset sepsis and its public health burden: a systematic review. Syst. Rev..

