# Supplementary material for: The effects of sociodemographic factors and comorbidities on sepsis: A nationwide Swedish cohort study
Source: Prev Med Rep. 2023 Jul 16;35:102326. doi: 10.1016/j.pmedr.2023.102326 (PMC10374593; doi:10.1016/j.pmedr.2023.102326)
Supplement: Supplementary data 1 [file mmc1.docx]

**Supplementary material**

**Table S1**. ICD-10 codes and number of sepsis cases in adults in Sweden 1997–2018

**Table S2.** Charlson Comorbidity Index (1992–1996) of adults with and without sepsis in Sweden

**Table S3.** Association of individual sociodemographic factors, comorbidities, and sepsis, after excluding aged 15–49 years individuals, in adults in Sweden 1997–2018

**Table S4.** Association of individual sociodemographic factors, comorbidities, and sepsis, follow-up in adults in Sweden 1997–2002

**Table S5.** Association of individual sociodemographic factors, comorbidities, and sepsis in adults in Sweden 1997–2018, without any exclusions (N=7 497 111)

| **Table S1**. ICD-10 codes and number of sepsis cases in adults in Sweden 1997–2018 | | | | | |
| --- | --- | --- | --- | --- | --- |
| **Type of Sepsis** | **ICD-10 code** | | **No.** | **%** |  |
| Streptococcal sepsis | A40 | | 21 395 | 13.2 |  |
| Other sepsis | A41 | | 131 209 | 81.2 |  |
| Severe sepsis | R65.1 | | 3964 | 2.5 |  |
| Septic shock | R57.2 | | 1801 | 1.1 |  |
| Sepsis (due to) (in) candidal | B37.7 | | 872 | 0.5 |  |
| Sepsis (due to) (in) herpesviral | B00.7 | | 26 | 0.0 |  |
| Sepsis (due to) (in) actinomycotic | A42.7 | | 31 | 0.0 |  |
| Sepsis (due to) (in) anthrax | A22.7 | | 2 | 0.0 |  |
| Sepsis (due to) (in) Erysipelothrix | A26.7 | | 29 | 0.0 |  |
| Sepsis (due to) (in) extraintestinal yersiniosis | | A28.2 | 37 | 0.0 |  |
| Sepsis (due to) (in) listerial | A32.7 | | 345 | 0.2 |  |
| Sepsis (due to) (in) melioidosis | A24.1 | | 5 | 0.0 |  |
| Sepsis (due to) (in) meningococcal | A39.2 | | 154 | 0.1 |  |
| Sepsis (due to) (in) meningococcal | A39.3 | | 5 | 0.0 |  |
| Sepsis (due to) (in) meningococcal | A39.4 | | 65 | 0.0 |  |
| Sepsis (due to) (in) plague | A20.7 | | 0 | 0.0 |  |
| Sepsis (due to) (in) tularemia | A21.7 | | 14 | 0.0 |  |
| Salmonella sepsis | A02.1 | | 1023 | 0.6 |  |
| Sepsis (due to) Shigella | A03.9 | | 193 | 0.1 |  |
| Sepsis (due to) Pasteurellosis | A28.0 | | 355 | 0.2 |  |
| Sepsis (due to) brucellosis | A23.9 | | 33 | 0.0 |  |
| Sepsis (due to) (in) gonococcal | A54.86 | | 0 | 0.0 |  |
| **All above** |  | | **161 558** | **100.0** |  |
| ICD-10: 10th revision of the International Classification of Diseases. No.: Number of cases. | | | | | |

| **Table S2.** Charlson Comorbidity Index (1992–1996) of adults with and without sepsis in Sweden | | | | | |  |
| --- | --- | --- | --- | --- | --- | --- |
|  | **The 9th revision of the International Classification of Diseases (ICD-9)** | **Without sepsis** | | **With sepsis** | | **Total events** |
| **Comorbidities** |  | No | % | No | % | No |
| Myocardial infarction | 410.x, 412.x | 71 271 | 94.7 | 4001 | 5.3 | 75 272 |
| Congestive heart failure | 398.91, 402.01, 402.11, 402.91, 404.01, 404.03. 404.11, 404.13, 404.91, 404.93, 425.4 - 425.9, 428.x. | 45 888 | 94.3 | 2783 | 5.7 | 48 671 |
| Peripheral vascular disease | 093.0, 437.3, 440.x, 441.x, 443.1 - 443.9, 447.1, 557.1. 557.9, V43.4. | 21 629 | 92.9 | 1644 | 7.1 | 23 273 |
| Cerebrovascular disease | 362.34, 430.x - 438.x. | 91 527 | 94.5 | 5335 | 5.5 | 96 862 |
| Dementia | 290.x, 294.1, 331.2. | 12 820 | 98.0 | 255 | 2.0 | 13 075 |
| Chronic pulmonary disease | 416.8, 416.9, 490.x - 505.x, 506.4, 508.1, 508.8. | 46 818 | 95.1 | 2403 | 4.9 | 49 221 |
| Rheumatic disease | 446.5, 710.0 - 710.4, 714.0 - 714.2, 714.8, 725.x. | 23 068 | 90.4 | 2440 | 9.6 | 25 508 |
| Peptic ulcer disease | 531.x - 534.x. | 25 274 | 94.5 | 1474 | 5.5 | 26 748 |
| Mild liver disease | 070.22, 070.23, 070.32, 070.33, 070.44, 070.54. 070.6, 070.9, 570.x, 571.x, 573.3, 573.4, 573.8, 573.9. V42.7. | 9784 | 93.0 | 733 | 7.0 | 10 517 |
| Diabetes without chronic complication | 250.0 - 250.3, 250.8, 250.9. | 34 817 | 91.6 | 3179 | 8.4 | 37 996 |
| Diabetes with chronic complication | 250.4 - 250.7. | 7569 | 88.1 | 1022 | 11.9 | 8591 |
| Hemiplegia or paraplegia | 334.1, 342.x, 343.x, 344.0 - 344.6, 344.9. | 2214 | 90.9 | 222 | 9.1 | 2436 |
| Renal disease | 403.01, 403.11, 403.91, 404.02, 404.03, 404.12. 404.13, 404.92, 404.93, 582.x, 583.0 - 583.7, 585.x. 586.x, 588.0, V42.0, V45.1, V56.x. | 4516 | 78.6 | 1228 | 21.4 | 5744 |
| Any malignancy, including lymphoma and leukemia, except malignant neoplasm of skin | 140.x - 172.x, 174.x - 195.8, 200.x - 208.x, 238.6. | 84 491 | 93.3 | 6037 | 6.7 | 90 528 |
| Moderate or severe liver disease | 456.0 - 456.2, 572.2- 572.8. | 598 | 87.2 | 88 | 12.8 | 686 |
| Metastatic solid tumor | 196.x - 199.x. | 1854 | 94.2 | 115 | 5.8 | 1969 |
| AIDS/HIV | 042.x - 044.x. | 89 | 85.6 | 15 | 14.4 | 104 |

| **Table S3.** Association of individual sociodemographic factors, comorbidities, and sepsis, after excluding aged 15–49 years individuals, in adults in Sweden 1997–2018 | | | |  |  |
| --- | --- | --- | --- | --- | --- |
| Covariates | HR* | 95% CI | | |  |
| **Age** (ref. age 50–59 years) |  |  |  | |  |
| 60–69 | 2.01 | 1.98 | 2.04 | |  |
| 70–79 | 3.58 | 3.52 | 3.63 | |  |
| ≥ 80 | 4.74 | 4.64 | 4.84 | |  |
| **Sex** (ref. women) | 1.82 | 1.80 | 1.84 | |  |
| **Educational level** (ref. > 12 years) |  |  |  | |  |
| ≤ 9 | 1.12 | 1.10 | 1.14 | |  |
| 10–12 | 1.05 | 1.03 | 1.07 | |  |
| **Family income** (ref. high) |  |  |  | |  |
| Low | 1.13 | 1.11 | 1.15 | |  |
| Middle | 1.12 | 1.11 | 1.14 | |  |
| **Region of residence** (ref. large cities) |  |  |  | |  |
| Southern Sweden | 0.79 | 0.78 | 0.80 | |  |
| Northern Sweden | 0.82 | 0.80 | 0.83 | |  |
| **Marital status** (ref. married/cohabiting) | 1.12 | 1.11 | 1.14 | |  |
| **Country of Origin** (ref. Sweden) |  |  |  | |  |
| Eastern Europe | 0.95 | 0.91 | 0.98 | |  |
| Western countries | 1.01 | 0.99 | 1.03 | |  |
| Middle East/North Africa | 1.01 | 0.94 | 1.10 | |  |
| Africa (excluding North Africa) | 0.84 | 0.67 | 1.07 | |  |
| Asia/Oceania (excluding Middle East) | 0.96 | 0.88 | 1.06 | |  |
| Latin America/Caribbean | 0.80 | 0.70 | 0.91 | |  |
| **Charlson Comorbidity Index** (ref. Low) |  |  |  | |  |
| Moderate (1–2 p) | 2.07 | 2.04 | 2.10 | |  |
| High (>2 p) | 3.04 | 2.32 | 3.99 | |  |
| **Severe mental disorders** (ref. No diagnosis) | 1.48 | 1.41 | 1.55 | |  |
| *: Fully adjusted. | | | | | |

| **Table S4.** Association of individual sociodemographic factors, comorbidities, and sepsis, follow-up in adults in Sweden 1997–2002 | | | |  |
| --- | --- | --- | --- | --- |
| Covariates | HR* | 95% CI | | |
| **Age** (ref. age 15–49 years) |  |  |  | |
| 50–59 | 1.38 | 1.36 | 1.41 | |
| 60–69 | 1.67 | 1.64 | 1.70 | |
| 70–79 | 2.54 | 2.50 | 2.58 | |
| ≥ 80 | 4.93 | 4.83 | 5.03 | |
| **Sex** (ref. women) | 1.17 | 1.16 | 1.18 | |
| **Educational level** (ref. > 12 years) |  |  |  | |
| ≤ 9 | 1.06 | 1.05 | 1.08 | |
| 10–12 | 0.99 | 0.97 | 1.00 | |
| **Family income** (ref. high) |  |  |  | |
| Low | 1.12 | 1.11 | 1.14 | |
| Middle | 1.08 | 1.07 | 1.10 | |
| **Region of residence** (ref. large cities) |  |  |  | |
| Southern Sweden | 0.98 | 0.97 | 0.99 | |
| Northern Sweden | 1.05 | 1.03 | 1.06 | |
| **Marital status** (ref. married/cohabiting) | 1.11 | 1.10 | 1.12 | |
| **Country of Origin** (ref. Sweden) |  |  |  | |
| Eastern Europe | 0.99 | 0.96 | 1.02 | |
| Western countries | 1.03 | 1.01 | 1.05 | |
| Middle East/North Africa | 0.99 | 0.94 | 1.05 | |
| Africa (excluding North Africa) | 1.13 | 1.01 | 1.28 | |
| Asia/Oceania (excluding Middle East) | 1.07 | 1.00 | 1.14 | |
| Latin America/Caribbean | 0.98 | 0.89 | 1.08 | |
| **Charlson Comorbidity Index** (ref. Low) |  |  |  | |
| Moderate (1–2 p) | 1.63 | 1.61 | 1.65 | |
| High (>2 p) | 1.53 | 1.22 | 1.93 | |
| **Severe mental disorders** (ref. No diagnosis) | 1.23 | 1.19 | 1.28 | |
| *: Fully adjusted. | | | |  |

| **Table S5.** Association of individual sociodemographic factors, comorbidities, and sepsis in adults in Sweden 1997–2018, without any exclusions (N=7 497 111) | | | |  |
| --- | --- | --- | --- | --- |
| Covariates | HR* | 95% CI | | |
| **Age** (ref. age 15-49 years) |  |  |  | |
| 50-59 | 3.84 | 3.78 | 3.90 | |
| 60–69 | 7.59 | 7.46 | 7.71 | |
| 70–79 | 13.25 | 13.04 | 13.47 | |
| ≥ 80 | 16.05 | 15.73 | 16.38 | |
| **Sex** (ref. women) | 1.62 | 1.60 | 1.64 | |
| **Educational level** (ref. > 12 years) |  |  |  | |
| ≤ 9 | 1.09 | 1.08 | 1.11 | |
| 10–12 | 1.04 | 1.02 | 1.05 | |
| **Family income** (ref. high) |  |  |  | |
| Low | 0.89 | 0.87 | 0.91 | |
| Middle | 1.10 | 1.09 | 1.11 | |
| **Region of residence** (ref. large cities) |  |  |  | |
| Southern Sweden | 0.83 | 0.82 | 0.84 | |
| Northern Sweden | 0.86 | 0.85 | 0.87 | |
| **Marital status** (ref. married/cohabiting) | 1.08 | 1.07 | 1.10 | |
| **Country of Origin** (ref. Sweden) |  |  |  | |
| Eastern Europe | 0.85 | 0.83 | 0.88 | |
| Western countries | 0.98 | 0.96 | 1.00 | |
| Middle East/North Africa | 0.67 | 0.64 | 0.70 | |
| Africa (excluding North Africa) | 0.72 | 0.65 | 0.79 | |
| Asia/Oceania (excluding Middle East) | 0.83 | 0.78 | 0.89 | |
| Latin America/Caribbean | 0.76 | 0.69 | 0.83 | |
| **Charlson Comorbidity Index** (ref. Low) |  |  |  | |
| Moderate (1–2 p) | 2.37 | 2.34 | 2.40 | |
| High (>2 p) | 4.43 | 3.56 | 5.50 | |
| **Severe mental disorders** (ref. No diagnosis) | 1.68 | 1.62 | 1.74 | |
| *: Fully adjusted. *Notes*: Missing values were in Table S5 not excluded. For family income, missing values were instead included in the lowest level of income; for Educational level, missing values were instead included in the lowest educational level; for Region of residence, missing values were instead included in large cities; and for country of origin, missing values were included in Sweden. | | | |  |
